# Diagnostic and Prognostic Value of Methyltransferase Like 13 in Hepatocellular Carcinoma Based on Bioinformatics Analysis

**DOI:** 10.5152/tjg.2024.22878

**Published:** 2024-05-01

**Authors:** Yuanzhang Wen, Jiwei Xu, Huancheng Zhou, Wanwei Liu

**Affiliations:** Department of Hepatobiliary Surgery, Meizhou People’s Hospital, Meizhou, Guangdong, China

**Keywords:** METTL13, hepatocellular carcinoma, bioinformatics analysis, prognostic value

## Abstract

**Background/Aims:**

Hepatocellular carcinoma (HCC) is one of the cancers with the highest incidence and mortality rates. This study aims to explore the diagnostic and prognostic utility of methyltransferase like 13 (METTL13) in patients with HCC via bioinformatics analysis.

**Materials and Methods:**

We obtained mRNA data of HCC from the database of the Cancer Genome Atlas (TCGA), drawing survival curve by R 4.2.1 software. Cox regression analysis was conducted based on tumor stage and METTL13 expression. The GSE114564 dataset was chosen from the Gene Expression Omnibus. The differences in serum METTL13 levels between the groups of early HCC (eHCC) and non-cancer controls were evaluated. Using a receiver operating characteristic curve, we calculated the area under the curve (AUC) of serum METTL13 for diagnosing eHCC.

**Results:**

A total of 225 cases with HCC were screened from TCGA, and 29 cases were normal controls. The results showed that the METTL13 expression in the HCC group was higher than that in the normal controls (*P* < .001). The univariate [hazard ratio (HR) = 1.895, *P *= .006] and multivariate Cox regression (HR = 1.702, *P *= .037) analyses showed that high METTL13 expression reduced overall survival in HCC. Serum METTL13 levels were higher in the eHCC group than in the non-cancer controls (*P *= .008). The optimum AUC for predicting eHCC by serum METTL13 was 0.7091.

**Conclusion:**

Serum METTL13 has a moderate diagnostic value for eHCC. High METTL13 expression is correlated with a worse prognosis in patients with HCC. Methyltransferase like 13 possesses the potential to be a novel biomarker for HCC.

**Main Points**

- Serum methyltransferase like 13 (METTL13) has a moderate diagnostic value for early hepatocellular carcinoma (HCC).
- High METTL13 expression is related to worse prognosis in HCC.
- This is the first study suggesting that METTL13 has the potential to serve as a novel biomarker for HCC.

## Introduction

Accounting for about 75%-85% of primary liver cancer, hepatocellular carcinoma (HCC) is one of the cancers with the highest incidence rate, which ranks fifth worldwide.^1^ Moreover, it is the fourth most common cancer-related cause of death in the world.^2^ Hepatocellular carcinoma is a high-grade malignant tumor. Even after successful radical resection, it still shows the characteristics of rapid invasive growth, early metastasis, and poor prognosis.^3,4^ Since the early symptoms of HCC are not typical and the lesions are small, it is challenging to detect early HCC (eHCC). When patients with HCC show symptoms, most of them are in the advanced stage and usually have a poor prognosis. Early detection of HCC is the most critical step in the treatment process. Therefore, it is important to identify diagnostic and prognostic markers for patients with tumors.

Methyltransferase like 13 (METTL13), also known as faint expression in normal tissues, aberrant overexpression in tumors (FEAT), localized at 1q24.3, was originally identified as an inhibitor of nuclear apoptosis in rat brains.^5^ Studies reported that METTL13 expression was upregulated in liver cancer,^6^ breast cancer, lung cancer,^7^ and head and neck cancers.^8^ Liu et al^9^ showed that METTL13-mediated K55 methylation of eukaryotic translation elongation factor-1A (eEF1A) promoted tumorigenicity. In addition, METTL13 inhibited the progression of clear cell renal cell carcinoma through phosphatidylinositol 3-kinase/protein kinase B (AKT)/mammalian target of the rapamycin/hypoxia-inducible factor-1 alpha pathway.^10^ In HCC, hematological and neurological expressed 1-like (HN1L)-mediated transcription axis activator protein 2 gamma/METTL13/transcription factor 3 zinc finger E-box-binding homeobox 1 has a driving effect on tumor growth and metastasis.^6^ As a possible therapeutic target for cancer, it is necessary to investigate the action of METTL13 in HCC. However, the significance of METTL13 in patients with HCC has not been explored.

Therefore, this study used the Cancer Genome Atlas (TCGA) and Gene Expression Omnibus (GEO) to elucidate the diagnostic value of serum METTL13 level in eHCC, as well as the prognostic function of METTL13 mRNA expression in cancer tissue for HCC, to identify potential targets for early diagnosis and treatment of HCC.

## Materials and Methods

### The Cancer Genome Atlas Database Download

The mRNA and clinical information of 225 HCC cases and 29 normal cases (paracancerous tissues) were obtained from the TCGA database (https://portal.gdc.cancer.gov/). The pathological type of all patients was HCC. The mRNA expression of METTL13 was screened out. Clinical data included pathological type, tumor-node-metastasis (TNM) stage, age, gender, and race. The function “merge” in R 4.2.1 software was utilized to merge the METTL13 mRNA expression data of HCC patients with corresponding clinical data. Inclusion criteria were as follows: the survival outcomes and survival time of HCC patients were provided in the clinical information.

### Survival Curve Drawing

Based on the median expression of METTL13 mRNA, the cases with HCC were divided into the following groups: groups of high METTL13 expression and low METTL13 expression. We selected the function “survival” in R 4.2.1 software to capture the survival curve between METTL13 expression and the prognosis of HCC patients.

### Analyses of Cox Regression

The age, race, TNM stage, gender, and METTL13 expression were entered into the Cox regression models of univariate and multivariate analyses. The hazard ratios (HRs), as well as 95% CIs and *P*-values, were estimated.

### Gene Expression Omnibus Database Download

The dataset GSE114564 was downloaded from the GEO database (https://www.ncbi.nlm.nih.gov/geo/query/acc.cgi?acc=GSE114564), containing the samples from 15 normal liver (NL), 20 chronic hepatitis (CH), 10 liver cirrhosis (LC), 10 dysplastic nodule (DN), 18 eHCC, and 45 advanced HCC. In this study, 45 advanced HCC cases were excluded, and NL, CH, LC, and DN were classified as the non-cancer control groups. The differences in serum METTL13 levels between the groups of non-cancer control and eHCC were assessed. Besides, we obtained the receiver operating characteristic (ROC) curve of METTL13 for diagnosing eHCC, calculating an area under the curve (AUC).

### Statistical Analysis

R 4.2.1 software was used to draw the survival curve. The ROC curve of serum METTL13 level in the diagnosis of eHCC was drawn by Stata 15.0 statistical software. The differences in age, gender, race, and TNM stage between the high and low METTL13 expression groups, the differences in serum METTL13 between the eHCC group and the non-cancer control group, and univariate and multivariate Cox regression analyses were performed using Statistical Package for the Social Sciences Statistics software 16.0 software (SPSS Inc., Chicago, Ill, USA). The difference in METTL13 expression between HCC and paracancerous tissue groups was statistically analyzed by 2 independent sample *t*-tests. As the age of the HCC group and the METTL13 level of the eHCC group did not meet normality, the difference in age between the high and low METTL13 expression groups and the difference in serum METTL13 between the eHCC and non-cancer control groups were processed by the Mann–Whitney *U*-test. The chi-square test was used to determine the differences in gender, race, and TNM stage between METTL13 high- and low-expression groups.

## Results

### Basic Information of the Hepatocellular Carcinoma Cases Based on the Cancer Genome Atlas

According to the inclusion criteria, 225 patients with HCC from TCGA were included in this research, comprising 225 HCC tissues and 29 paracancerous tissues (normal control). The basic features of the eligible subjects are shown in Table 1. The included HCC patients were mainly White and Asian, accounting for 92.89% (209 out of 225). Males accounted for 64.89% (146 out of 225), and all patients covered the TNM I-IV stage (Table 1).

### Association of Methyltransferase Like 13 mRNA Expression with Clinicopathological Features of Hepatocellular Carcinoma Patients

The median expression of METTL13 was 1669. No significant differences existed in age, gender, race, and tumor stage between the high and low METTL13 mRNA expression groups in HCC patients (Table 2) (all *P *> .05).

### Differential Expression of Methyltransferase Like 13 in Hepatocellular Carcinoma Patients

**Figure 1.**
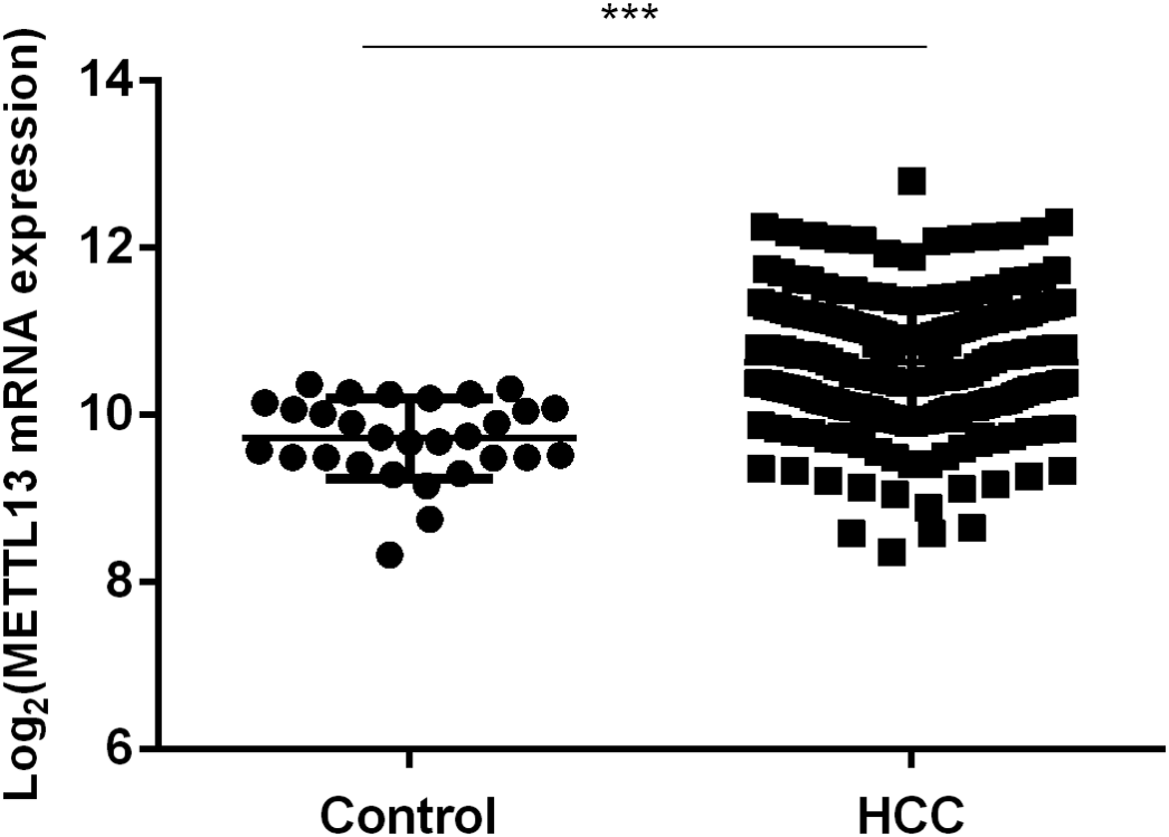
Differential expression of METTL13 mRNA in HCC patients. ***, represents *P* < .001. Control: paracancerous tissues. HCC, hepatocellular carcinoma; METTL13, methyltransferase like 13.

**Figure 2.**
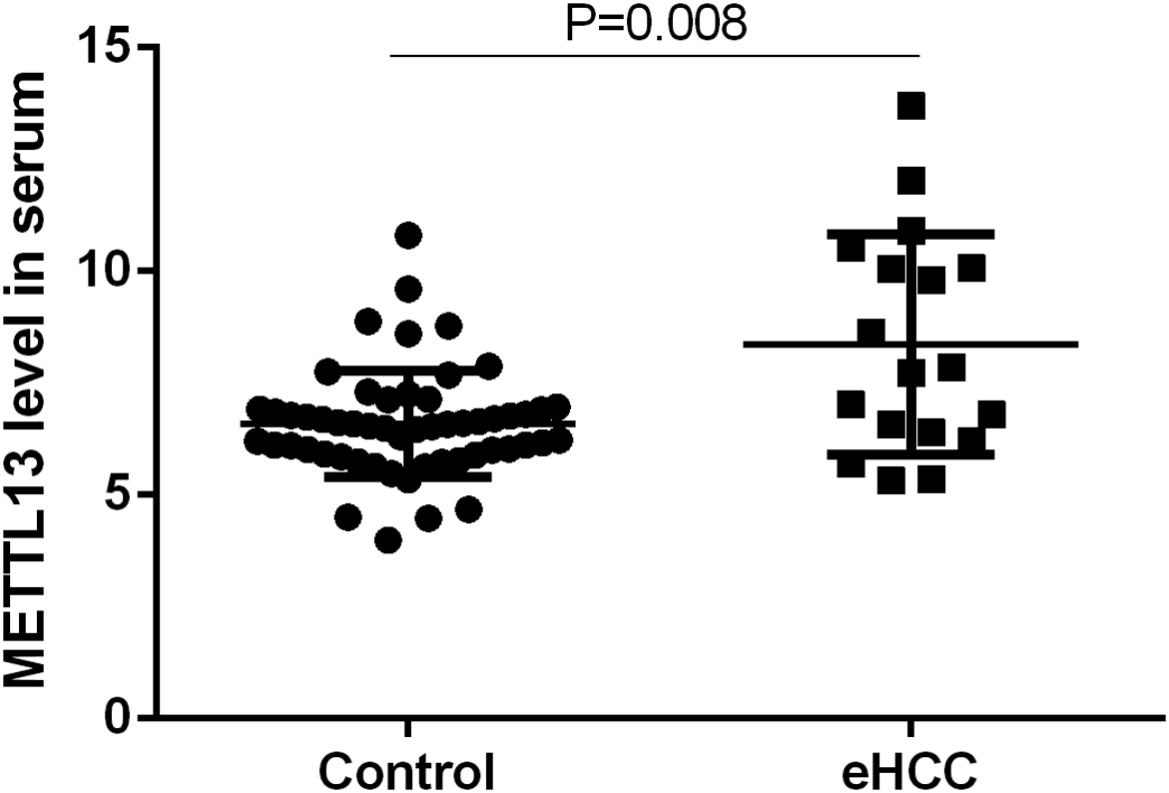
Differential expression of METTL13 level in eHCC patients. eHCC, early hepatocellular carcinoma; METTL13, methyltransferase like 13.

The gene expression data of METTL13 obtained from TCGA were processed on a logarithmic scale with 2 as the substrate number. The expression of METTL13 mRNA in the HCC group was higher compared to the expression in normal control (Figure 1) (*P* < .001). Compared to the non-cancer control group (Figure 2), the METTL13 level was increased in the eHCC group (*P *= .008).

### Correlation Between Methyltransferase like 13 and Prognosis of Hepatocellular Carcinoma Patients

**Figure 3.**
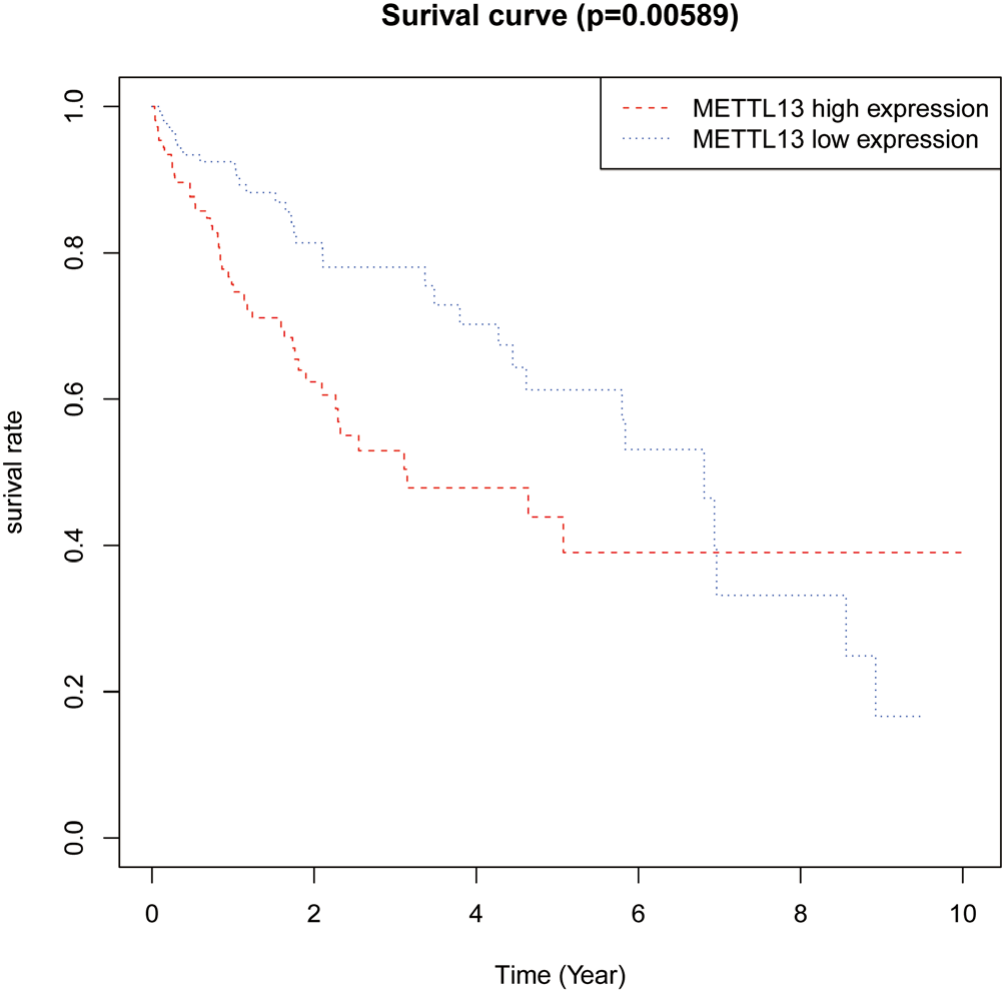
Correlation between METTL13 expression and OS in HCC patients. HCC, hepatocellular carcinoma; METTL13, methyltransferase like 13; OS, overall survival.

As shown in Figure 3, HCC patients with a high expression of METTL13 mRNA had lower 5-year overall survival (OS) rates compared to patients with low expression of METTL13 mRNA (*P* < .01).

### Cox Regression Analysis

According to univariate Cox regression analysis (Table 3), age, race, and gender were not found to be correlated with the prognostic value of HCC patients (all *P* > .05). However, tumor stage and METTL13 expression were correlated with the prognosis in HCC patients. The survival outcomes worsened as the tumor stage increased (HR = 1.556, 95% CI: 1.191~2.035,* P *= .001). The prognosis of HCC patients with high METTL13 expression was worse than those with low METTL13 expression (OS: HR = 1.895, 95% CI: 1.194~3.008, *P *= .006). The findings of multivariate Cox regression analysis were consistent with those of univariate results. Based on multivariate Cox regression analysis, compared with the low expression of METTL13, the high expression of METTL13 reduced the OS of HCC patients by 1.702 times (HR = 1.702, 95% CI: 1.032~2.806, *P *= .037). Therefore, the METTL13 expression significantly influences the prognosis of HCC patients.

### Diagnostic Value of Serum Methyltransferase like 13 for Early Hepatocellular Carcinoma

**Figure 4.**
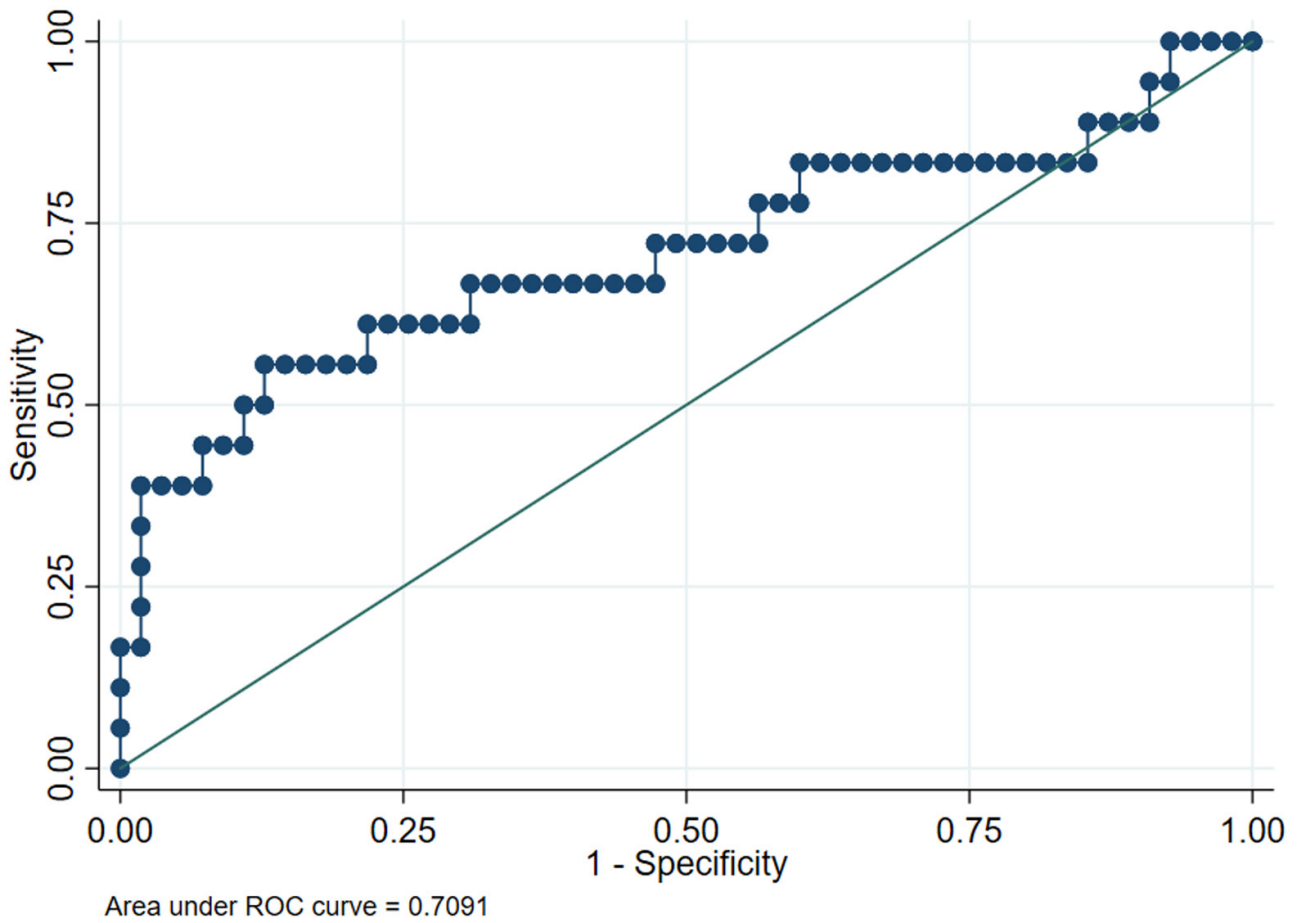
Diagnostic value of serum METTL13 for eHCC. eHCC, early hepatocellular carcinoma; METTL13, methyltransferase like 13; ROC, receiver operating characteristic.

The ROC curve (Figure 4) showed that serum METTL13 had a moderate diagnostic performance for eHCC diagnosis. When the cutoff value was 7.704, the optimum AUC was 0.7091 (95% CI: 0.5485~0.8679).

## Discussion

Serum tumor marker detection is a non-invasive, objective, convenient, and reproducible assessment method. Compared with imaging detection, it is relatively cost effective and, therefore has high patient compliance.^11^ Alpha-fetoprotein (AFP) is widely used in the clinical diagnosis of HCC, but its diagnostic value remains to be discussed.^12,13^ Alpha-fetoprotein can be increased in patients with pregnancy, chronic or active liver disease, and gonadal embryo-derived tumors,^14^ which will also reduce the efficiency of AFP for eHCC screening. Therefore, it is essential to find a new serological marker to screen for eHCC and improve the survival of patients.

Methyltransferase like 13 can promote the development and evolution of HCC in a variety of ways, but its mechanism in the occurrence and development of HCC is still not completely clear. In this research, the METTL13 expression in HCC was analyzed by the bioinformatics method, and the diagnostic value, as well as the prognosis assessment of METTL13 in HCC patients, was preliminarily evaluated and discussed. This research presented that the expression of METTL13 mRNA was upregulated in HCC tissues, and the higher METTL13 mRNA expression was correlated with poor survival outcomes of patients with HCC. Cox regression indicated that METTL13 expression might be an independent prognostic factor for patients with HCC, both in the univariate and in the multivariate analyses. At the same time, predictive analysis of the GSE114564 dataset showed that serum METTL13 level had a moderate diagnostic accuracy for eHCC, with an AUC of more than 70%. Additionally, METTL13 mRNA was upregulated in the HCC tissues compared to adjacent tissues. In serum samples, METTL13 levels were higher in the HCC group compared to the non-cancer controls. Besides, in the serum sample research, the non-cancer control group included NL, CH, LC, and DN, while HCC was restricted to the early stage, with advanced HCC excluded. Therefore, METTL13 as a serum marker can be used as a significant supplement to AFP in the diagnosis of eHCC, which has critical clinical value.

Recently, several studies have explored the role of METTL13 in cancer. Wu et al^15^ showed that METTL13 promoted the growth and metastasis of gastric cancer cells through the eEF1A/HN1L-positive feedback loop. For most cancers, METTL13 is upregulated. However, some cancers showed different results. Zhang et al^16^ showed that METTL13 was downregulated in bladder cancer and inhibited cell proliferation, migration, and invasion. Currently, the mechanism of METTL13 in cancer is still not clearly understood, and further studies are needed to explore it.

In conclusion, this study mainly used bioinformatics analysis to clarify the expression of METTL13 mRNA in HCC. The results indicated that METTL13 could be adopted as a significant biomarker for the clinical prognosis of patients with HCC and the diagnosis of eHCC. The findings of this research provide a new basis for METTL13 to become a new diagnostic and prognostic marker for HCC. As the above research results are all derived from big data analysis, they have certain limitations. Based on this, it is essential to further verify and elaborate the role and related molecular mechanism of METTL13 in HCC tissues from clinical samples and basic experiments in the future in order to complement and improve the systematic research on METTL13.

## Figures and Tables

**Table 1.** Characteristics of the Cancer Genome Atlas–Liver Hepatocellular Carcinoma Cohort

Characteristics	Data
Overall, n	225
Age (years) (mean ± SD)	58.53 ± 13.88
Race, n	
Asian	98
White	111
Others	12
NR	4
Gender, n	
Male	146
Female	79
TNM stage, n	
I	110
II	48
III-IV	51
NR	16

Others: Black or African American and American Indian or Alaska native.

NR, not reported; TNM, tumor–node–metastasis.

**Table 2.** The Correlation of Methyltransferase Like 13 Expression with the Clinical Pathological Characteristics of the Cancer Genome Atlas–Liver Hepatocellular Carcinoma Cohort

Characteristics	METTL13 Low Expression	METTL13 High Expression	*P*
Age (years) (mean ± SD)	57.12 ± 13.79	59.96 ± 13.88	.129^#^
Gender, n (%)			
Male	79 (69.9)	67 (59.8)	.113
Female	34 (30.1)	45 (40.2)	
Race, n (%)			
Asian	47 (42.7)	51 (45.9)	.778
White	56 (50.9)	55 (49.5)	
Others	7 (6.4)	5 (4.5)	
TNM stage, n (%)			
I	62 (57.4)	48 (47.5)	.285
II	24 (22.2)	24 (23.8)	
III-IV	22 (20.4)	29 (28.7)	

Others: Black or African American and American Indian or Alaska native.

LIHC, liver hepatocellular carcinoma; METTL13, methyltransferase like 13; TNM, tumor-node-metastasis.

**Table 3.** Univariate and Multivariate Analysis of Overall Survival in the Cancer Genome Atlas by Cox Regression Model

Characteristics	Number		Univariate Analysis			Multivariate Analysis		
	Patients	Events	HR	95%CI	*P*	HR	95%CI	*P*
Age (years)	-	-	1.006	0.989~1.024	.471	1.011	0.991~1.030	.278
Race								
Asian (Ref.)	98	28	1.000			1.000		
White	111	42	1.082	0.664~1.763	.752	0.811	0.464~1.417	.461
Others	12	4	1.327	0.465~3.788	.598	1.979	1.0.593~6.604	.267
TNM stage								
I	110	27	1.556	1.191~2.035	**.001**	1.562	1.169~2.086	**.003**
II	48	17						
III-IV	51	25						
Gender								
Male	146	42	1.000			1.000		
Female	79	34	1.426	0.905~2.246	.126	1.416	0.832~2.408	.200
METTL13 expression								
Low	113	32	1.000			1.000		
High	112	44	1.895	1.194~3.008	**.006**	1.702	1.032~2.806	**.037**

Others: Black or African American and American Indian or Alaska native.

HR, hazard ratio; METTL13, methyltransferase like 13; Ref., reference; TNM, tumor-node-metastasis.
